# Quality of life of prosthetic and orthotic users in South India: a cross-sectional study

**DOI:** 10.1186/s12955-019-1116-y

**Published:** 2019-03-20

**Authors:** Lina Magnusson, Ritu Ghosh, Katrine Randbøll Jensen, Katharina Göbel, Jenny Wågberg, Sofia Wallén, Alma Svensson, Rebecka Stavenheim, Gerd Ahlström

**Affiliations:** 1 Department of Health Sciences, Faculty of Medicine, Lund University, Box 157, 221 00 Lund, Sweden; 2 Mobility India Rehabilitation Research and Training Centre, 1st and 1st A Cross, Phase II, J.P. Nagar, Bangalore, Karnataka e-560078 India

**Keywords:** Assistive technology, Community-based rehabilitation, Disability, India, Low-income country, Orthosis, Prosthesis, Quality of life, WHOQOL-Bref

## Abstract

**Background:**

The aim of this study was to compare QOL among people in India using lower-limb prostheses or orthoses with people without disability. A further aim was to compare subgroups and investigate whether QOL was associated with physical disability, gender, income, living area, and education.

**Methods:**

A cross-sectional questionnaire study in which the World Health Organization Quality of Life-Bref was used to collect self-reported data. A total of 277 participants from India were included, 155 with disability and 122 without. Group comparisons were conducted using the Mann–Whitney U and the Kruskal–Wallis tests and associations were explored using regression analyses of the four QOL domains: physical health, psychological, social relationships, and environment.

**Results:**

Participants with physical disability scored significantly lower than did participants without disability in three of the four QOL domains, i.e., physical health, (Median 14.29 vs 16.29; *p* < .001) psychological, (Median 14.67 vs. 15.33; *p* = **.**017) and environment **(**Median13.00 vs 14.00; *p* = .006). For people with disability those with no or irregular income and those not attending school having the lowest QOL scores in all four domains. Education was associated with all four QOL domains and income was associated with psychological and environment. Living in urban slums was associated with a higher risk of lower QOL in three QOL domains, i.e., physical health, psychology, and environment.

**Conclusions:**

Despite rehabilitation services, people with physical disability experienced lower QOL in terms of the physical health, psychological, and environmental domains than did people without disability. Community-based rehabilitation programmes for prosthetic and orthotic users need to increase and improve their rehabilitation services to increase income and improve access to education. Priority could be given to those who have no or irregular income, live in urban slums, and have not attended school to further improve their QOL.

## Background

Vulnerable groups with disability are seldom prioritized in low and middle income countries, these countries also often have high prevalence of communicable diseases such as poliomyelitis, but also injury, and accident that can cause disabilities. One way to understand the needs of people with physical disability people and assess the outcomes of a rehabilitation service is to measure those people’s quality of life (QoL) [1]. However, few studies [2–6] have compared the QOL of people living with and without disability or have addressed the QoL of people who require assistive devices to maintain mobility [7] in low- and middle-income countries. In India, Mankar et al. [2] found lower QOL in people with leprosy than in a control group, with women experiencing significantly lower QOL in the psychological domain and men significantly lower QOL in the physical and environmental domains than the control group. Another study from India [6] found lower QOL in a group of lower-limb amputees than in the general population, demonstrating that physical and mental health were lower in amputees than in the general population.

In a lower- to middle-income country such as India, many people with disability often lack access to the rehabilitation and assistive devices they need. According to WHO (World Health Organization), only one in ten people have access to needed assistive products [8]. Lower-limb assistive devices provide increased mobility, and positive correlation has been shown between mobility and quality of life [9]. A Tanzanian study using the EQ-5D questionnaire indicated poor health-related QOL due to a high rate of post-operative complications among amputees and extremely limited access to prosthetics [10]. It is important that people with disability have the opportunity to access education, employment, health and social services [11, 12], and assistive devices, all of which help people achieve better conditions [8, 13]. According to the World Report on Disability [1], QOL is “an individual’s perception of their position in life in the context of the culture and value systems in which they live and in relation to their goals, expectations, standards and concerns” [1] (p. 307). QOL is a broad concept encompassing a person’s physical health, psychological state, level of independence, social relationships, and personal beliefs as well as the environmental factors that affect a person [1].

Community-based rehabilitation (CBR) has been developed to provide opportunities, rights, and access for all people with disability equal to those of other citizens in the community, regardless of age, gender, type of disability, religion, and socioeconomic status. A study found that Cambodians with disability with no access to rehabilitation services had lower QOL than did those who received one of three categories of rehabilitation, i.e., prosthetics/orthotics with physiotherapy, CBR, or labour market assistance [14]. In that study, employment status and use of assistive devices were identified as important factors affecting QOL.

In India, official statistics indicate that 2.2% of the general population are people with disability, 20% of whom have a movement disability [15]. A physical disability can make it difficult to perform daily activities and earn income. Other problems often associated with disability and that influence QOL are discrimination and exclusion from social activities and society [14, 16]. A previous study found that various subgroups of people living with disability, i.e., the poorest, the least educated, older people, and women, often report significantly lower QOL [14]. Therefore, it is important to know about the living conditions that make people with disability most vulnerable and in need of special support. We expect gender, use of mobility assistive devices, area of living, and socioeconomic aspects such as education and income level are related to differences in perceived QOL, depending on whether people have a disability.

### Aim

The aim of this study was to compare QOL among people in India using lower-limb prostheses or orthoses with people without disability. A further aim was to compare subgroups and investigate whether QOL was associated with physical disability, gender, income, living area, and education.

## Method

### Design

A cross-sectional study design based on a questionnaire was employed. Self-reported QOL data from prosthetic and orthotic users and from a control group without disabilities were included. The term “assistive device” refers to prostheses and orthoses in this study, even if participants also used other devices, such as wheelchairs and crutches.

### Settings

This study was carried out at Mobility India, a non-governmental organization in Bengaluru, Karnataka state, India. Mobility India offers prosthetic and orthotic services, physiotherapy/occupational therapy services, and CBR programmes in slums and rural areas. Since 1994 CBR programmes for people with disability have been intended to improve the QOL of people with disability by focusing on their basic needs and ensuring their inclusion and participation in society. QOL outcome evaluations have been suggested as a way to evaluate rehabilitation services of CBR programmes [13]. The prosthetic and orthotic services and similar services were offered at rehabilitation centre and at Mobility India’s CBR project sites: 23 urban slums in Bangalore, 44 villages in Anekal Taluk rural district in Bangalore, and 225 villages in Chamrajnagar rural district in Karnataka.

### Sampling and participants

Participants with and without disability in the state of Karnataka, India, were selected to represent different types of living areas, i.e., non-slum urban areas, urban slums, and rural areas. Participants were eligible if they were 15–60 years old and spoke English and/or Kannada.

Participants were included in the disability group if they had a lower-limb physical disability and had received prosthetic and/or orthotic services within the last 3 years from Mobility India. Prostheses are used to replace the whole limb or a part thereof, while orthoses are applied to the limb to support or correct its function [17]. Exclusion criteria were blindness, deafness, severe cognitive disability, or a severe mental disorder. Staff or students from Mobility India using prosthetic and orthotic devices were not included in the study.

Participants were identified from Mobility India’s register and in collaboration with a network of CBR workers. Participants in the non-slum urban areas were contacted while they were visiting the Mobility India centre; participants in urban slums and rural areas were contacted through home visits.

The group without disability was selected for distributions of residence area, gender distribution, average age, and age range similar to the study group. To avoid bias in the QOL results, the group without disability excluded potential participants who had a close relative with disability.

Of the 285 people included, there were 8 dropouts due to incomplete questionnaires or exclusions. Reasons for dropping out were insufficient language skills in Kannada (2), cognitive disability (3), and started but did not want to complete the questionnaire (3).

### Measurements

Questions about demographics and characteristics were formulated for this study (see Tables 1 and 2). The World Health Organization Quality of Life-Bref (WHOQOL-Bref) questionnaire [18] is an abbreviated version of the WHOQOL-100 [19] addressed to adults. The instrument consists of two general question (one covering overall QOL and one the person’s general health situation) and 24 specific questions covering four domains of self-assessed QOL: physical health (seven items), psychological (six items), social relationships (three items), and environment (eight items) [18]. Each item is answered on a scale of 1 to 5, with 5 signifying the highest QOL. The English and Kannada versions of the WHOQOL-Bref were used in this study. The English version has displayed good criterion and content validity as well as test–retest reliability. The questionnaire also has good internal consistency [18]. The Kannada version of the questionnaire was translated during earlier research into QOL and HIV [20, 21] in collaboration with the WHOQOL group. The QOL assessment used a recommended time frame of 4 weeks [22]. Permission to use the English and Kannada versions of the WHOQOL-Bref [18] questionnaire in this study was provided by WHO. Cronbach’s alpha results for the four domains in this study were: physical health 0.75, psychological 0.66, social relationships 0.57, and environment 0.72.

**Table 1:** Characteristics of the study groups

	Total *N* (%)	Study group with disability, *n* (%)	Study group without disability, *n* (%)
Total	277 (100)	155 (55)	122 (45)
Gender			
Male	141 (51)	79 (51)	62 (51)
Female	136 (49)	76 (49)	60 (49)
Income			
No or Irregular	145 (52)	92 (59)	53 (43)
Yes, regular	132 (48)	63 (41)	69 (57)
Area of residence			
Non-slum urban	77 (28)	38 (24)	39 (32)
Urban slum	103 (37)	63 (41)	40 (33)
Rural	97 (35)	54 (35)	43 (35)
Education			
Not attended school	29 (12)	22 (18)	7 (6)
Attended school ≤10 years	99 (41)	50 (41)	49 (40)
Attended school ≥10 years	115 (47)	49 (41)	66 (54)

**Table 2:** Description of the study group with disability (*n* = 155)

	*n* (%)
Cause of disability	
Poliomyelitis	103 (67)
Accident	16 (10)
Gangrene	10 (7)
Cerebral palsy	5 (3)
Stroke	5 (3)
Congenital dislocation of hip	3 (2)
Burns	2 (1)
Spina bifida	2 (1)
Diabetes	2 (1)
Clubfoot	1 (1)
Other	6 (4)
Type of assistive device used	
Trans-tibial prosthesis users	19 (12)
Trans-femoral prosthesis users	11 (7)
Shoe rise users	8 (5)
Ankle-foot orthosis users	31 (20)
Knee-ankle-foot orthosis users	86 (56)
Other additional assistive devices	
Crutches	29 (19)
Wheelchair	5 (3)

### Data collection

Questions were administered face-to-face and read to all participants in Kannada or English by a trained research assistant or one of the authors (i.e., KRJ, KG, SW, JW, AS, and RS). Data were collected in the homes of participants with and without disability in the slums and rural areas and at Mobility India rehabilitation centres, on the streets, in markets, or at the sports arena in the non-slum urban group.

### Data analysis

Descriptive statistics were calculated as means and standard deviations of WHOQOL-Bref scores according to the manual; these scores range from 4 to 20 [22]. All data were tested for normality using the Shapiro–Wilk test, and non-parametric statistics were used as the data were not normally distributed. The Mann–Whitney U (two sided) and the Kruskal–Wallis tests were used to compare the groups with and without disability, and to compare the subgroups of gender (i.e., female vs. male), income (i.e., no or irregular income vs. regular income), area of residence (i.e., non-slum urban, urban slum, and rural areas), education (i.e., years in school), and type of device (i.e., prosthetics and orthotics). For statistical testing, the significance level was set to *α* ≤ 0.05.

Medians were calculated for each of the four QOL domains including all cases (*N* = 277), as follows: physical health 15.43, psychological 14.67, social relationships 16.00, and environment 13.50. Variables were dichotomized using the median for each domain as the cut-off and logistic regression analysis was conducted to explore which groups were associated with a risk of low QOL outcome in the four QOL domains. Univariate logistic regression was performed on the group with physical disability versus the group without disability using dichotomized variables for each domain. Furthermore, logistic regression adjusted for gender, income, area of residence, and education was performed (Table 6). Multivariate logistic regression was conducted to identify subgroups with lower QOL (Table 6). All data analysis was conducted using IBM SPSS Statistics 20.

## Results

### Characteristics of the study groups

Of the 277 participants in the total study group, the 155 with a disability (see Table 1) had an average age of 30.8 years (range 15–60 years, SD 11.17). The group without disability consisted of 122 participants with an average age of 30.7 years (range 16–60 years, SD 9.98). The mean duration of school attendance was significantly lower in the group with disability, i.e., 8.8 years (SD 5.2) versus 11.3 years (SD 5.0) in the group without disability (*p* = 0.001). Table 2 presents the participants’ cause of disability and type of assistive device.

### QOL of participants with and without disability

The scores for the general QOL were 14.37 in the group with disability and 14.75 in the group without (*p* = 0.464) and for general health, 14.89 in the group with disability were and 15.51 in the group without (*p* = 0.164). Table 3 shows the QOL results for the four domains and 26 single items included in the WHOQOL-Bref questionnaire. QOL was lower among those with disability in the physical health, psychological, and environmental domains. Participants with disability reported significantly lower QOL scores than people without disability on 11 of 26 items. The results indicated no significant differences in overall QOL, general health, and the social relationships domain between participants with disability and those without.

**Table 3:** Total scores for domains and single items included in WHOQOL-Bref and comparisons between the group with disability and the group without disability

	Group with disability, median (*n* = 155)	Group with disability, mean (95% CI) (*n* = 155)	Group without disability, median (*n* = 122)	Group without disability, mean (95% CI) (*n* = 122)	*P* value, Mann–Whitney U
General QOL	16.00	14.37 (13.76 to15.00)	16.00	14.75 (14.16 to 15.35)	.464
General health	16.00	14.89 (14.29 to 15.49)	16.00	15.51 (14.88 to 16.14)	.164
**Physical health, total score**	**14.29**	**14.29 (13.83 to 14.75)**	**16.29**	**16.16 (15.78 to 16.54)**	<.001*
Dependence on medication to function in everyday life	**12.00**	**13.39 (12.72 to 14.06)**	**16.00**	**15.64 (14.88 to 16.40)**	<.001*
Pain prevents you from doing what you need to do	**16.00**	**15.33 (14.57 to 16.08)**	**20.00**	**17.41 (16.72 to 18.10)**	<.001*
Enough energy for everyday life	**16.00**	**14.50 (13.83 to 15.19)**	**16.00**	**15.74 (15.07 to 16.41)**	.020*
Mobility, ability to get around	**12.00**	**13.42 (12.73 to 14.11)**	**16.00**	**15.87 (15.19 to 16.55)**	<.001*
Satisfaction with sleep	16.00	15.02 (14.33 to 15.71)	16.00	15.41 (14.72 to 16.10)	.599
Satisfaction with ability to perform daily living activities	**16.00**	**15.02 (14.41 to15.63)**	**16.00**	**16.49 (15.94 to 17.04)**	.001*
Satisfaction with capacity for work	**16.00**	**15.05 (14.41 to 15.68)**	**16,00**	**16.59 (16.01 to 17.16)**	.002*
**Psychological, total score**	**14.67**	**14.36 (13.94 to 14.79)**	**15.33**	**15.09 (14.69 to 15.50)**	.017*
Enjoyment of life	16.00	14.27 (13.57 to 14.97)	16.00	14.43 (13.76 to 15.10)	.960
To what extent do you feel your life to be meaningful	**12.00**	**13.70 (12.97 to 14.43)**	**16.00**	**15.15 (14.41 to 15.89)**	.007*
Ability to concentrate	16.00	15.51 (14.87 to 16.15)	16.00	15.21 (14.51 to 15.91)	.381
Accept of bodily appearance	16.00	13.89 (13.16 to 14.61)	16.00	14.75 (14.00 to 15.51)	.143
Satisfied with yourself	16.00	15.85 (15.27 to 16.42)	16.00	16.49 (15.92 to 17.06)	.164
Negative feelings, despair, and depression	16.00	13.57 (12.94 to 14.21)	16.00	14.52 (13.83 to 15.22)	.058
**Social relationships, total score**	16.00	14.62 (13.99 to 15.24)	16.00	15.56 (14.98 to 16.13)	.080
Satisfied personal relationships	16.00	14.50 (13.68 to 15.33)	16.00	15.77 (14.97 to 16.57)	.060
Satisfied with sex life	16.00	14.46 (13.27 to 15.65)	16.00	16.32 (15.52 to 17.12)	.072
Satisfied with support from friends	16.00	14.60 (13.82 to 15.37)	16.00	14.68 (13.88 to 15.49)	.925
**Environment, total score**	**13.00**	**12.82 (12.40 to 13.25)**	**14.00**	**13.76 (13.33 to 14.19)**	.006*
Safety and security in daily life	**16.00**	**13.78 (13.02 to 14.55)**	**16.00**	**15.05 (14.29 to 15.81)**	.035*
Healthy physical environment	12.00	13.11 (12.40 to 13.82)	12.00	13.71 (12.97 to 14.47)	.291
Enough money to meet your needs	**8.00**	**9.52 (8.80 to 10.24)**	**12.00**	**11.25 (10.43 to 12.06)**	.002*
Availability of information needed in everyday life	12.00	12.16 (11.48 to 12.83)	12.00	13.15 (12.38 to 13.93)	.072
Opportunity for leisure activities	12.00	12.52 (11.78 to 13.27)	12.00	11.77 (10.94 to 12.60)	.156
Satisfaction with conditions of your living place	16.00	14.37 (13.67 to 15.07)	16.00	15.28 (14.56 to 16.00)	.098
Satisfaction with access to health services	**16.00**	**13.68 (12.98 to 14.37)**	**16.00**	**14.82 (14.16 to 15.48)**	.032*
Satisfied with transportation	**16.00**	**13.58 (12.78 to 14.38)**	**16.00**	**15.08 (14.32 to 15.85)**	.016*

*Significant difference, *p* < 0.05

### Differences in QOL in relation to gender, income, area of residence, and education

Table 4 shows the comparisons between the group with disability and the group without. Men with disability reported significantly lower QOL scores than men without in physical health, psychological, and environmental domains. Women with disability reported significantly lower QOL scores than women without only in the physical health domain.

**Table 4:** Comparisons of quality of life in groups with and without disability by gender, income, area of residence, and education

	Physical health			Psychological			Social relationships			Environmental		
	Group with disability Mean (95% CI)	Group without disability Mean (95% CI)		Group with disability Mean (95% CI)	Group without disability Mean (95% CI)		Group with disability Mean (95% CI)	Group without disability Mean (95% CI)		Group with disability Mean (95% CI)	Group without disability Mean (95% CI)	
Gender												
Men with disability, *n* = 79, Men without disability, *n* = 62	**14.57 (13.96 to 15.18)**	**16.78 (16.26 to 17.31)**	<.001*	**14.57 (14.00 to 15.13)**	**15.49 (14.91 to 16.08)**	.014*	14.72 (13.88 to 15.56)	16.01 (15.27 to 16.75)	.054	**13.18 (12.63 to 13.74)**	**14.08 (13.49 to 14.68)**	.035*
Women with disability, *n* = 76 without disability, *n* = 60	**14.08 (13.41 to 14.77)**	**15.52 (15.02 to 16.03)**	.002*	14.22 (13.58 to 14.86)	14.68 (14.11 to 15.24)	.319	14.52 (13.58 to 14.45)	15.09 (14.20 to 15.98)	.557	12.45 (11.87 to 13.15)	13.43 (12.80 to 14.06)	.054
Income levels												
Group with regular income with disability, *n* = 63 without disability, *n* = 69	**15.17 (14.56 to 15.79)**	**16.27 (15.75 to 16.78)**	.009*	15.25 (14.68 to 15.82)	15.45 (14.96 to 15.94)	.465	15.82 (15.02 to 16.62)	15.50 (14.73 to 16.27)	.472	13.82 (13.24 to 14.39)	13.87 (13.32 to 14.43)	.768
Group without regular income with disability, *n* = 92 without disability, *n* = 53	**13.68 (13.14 to 14.37)**	**16.03 (15.46 to 16.60)**	<.001*	13.75 (13,24 to 14.37)	14.63 (13.95 to 15.31)	.062	**13.78 (12.92 to 14.65)**	**15.64 (14.73 to 16.54)**	.009*	**12.14 (11.63 to 12.75)**	**13.61 (12.92 to 14.31)**	.004*
Area of residence												
Group: non-slum urban with disability, *n* = 38 without disability, *n* = 39	15.01 (14.06 to 15.95)	15.79 (15.17 to 16.42)	.379	15.04 (14.31 to 15.76)	14.87 (14.16 to 15.58)	.629	15.46 (14.38 to 16.53)	16.22 (15.15 to 17.30)	.283	13.58 (12.74 to 14.42)	14.17 (13.40 to 14.95)	.450
Group urban slum with disability, *n* = 63without disability, *n* = 40	**13.77 (13.01 to 14.55)**	**16.37 (15.64 to 17.11)**	<.001*	**13.95 (13.33 to 14.73)**	**14.97 (14.25 to 15.69)**	.040*	14.39 (13.26 to 15.54)	15.75 (14.80 to 16.70)	.219	**12.23 (11.48 to 12.98)**	**13.94 (13.11 to 14.77)**	.002*
Group: rural with disability, *n* = 54 without disability, *n* = 43	**14.50 (13.82 to 15.18)**	**16.30 (15.68 to 16.94)**	<.001*	14.37 (13.62 to 15.13)	15.41 (14.68 to 16.14)	.057	14.28 (13.32 to 15.25)	14.78 (13.77 to 15.78)	.580	13.06 (12.46 to 13.65)	13.22 (12.56 to 13.88)	.765
Education												
Group, school ≤10 years with disability*, n* = 50without disability, *n* = 49	**14.49 (13.89 to 15.39)**	**16.05 (15.41 to 16.69)**	.004*	14.36 (13.68 to 15.25)	14.98 (14.31 to 15.65)	.158	15.34 (14.36 to 16.33)	15.35(14.45 to 16.24)	.971	12.97 (12.34 to 13.81)	13.29 (12.61 to 13.96)	.386
Group school > 10 years with disability, *n* = 49 without disability, *n* = 66	15.78 (15.17 to 16.39)	16.32 (15.86 to 16.78)	.197	15.76 (15,24 to 16.17)	15.10 (14.55 to 15.65)	.090	16.76 (16.06 to 17.46)	15.79 (15.01 to 16.57)	.084	13.99 (13.46 to 14.52)	14.03 (13.45 to 14.62)	.993

Note: The group without disability that had not attended school was excluded due to small sample (n = 7)

Comparing those with and those without disability who had no or irregular income (Table 4) identified differences in three of the four domains: physical health, social relationships, and environment. People with regular income and disability had lower QOL scores in the physical health domain than people with regular income and no disability. Differences were also found in all four domains within the group with disability between those who had regular income and those who did not.

People with disability in the urban slums and rural areas scored lower in the physical health domain than did people without (Table 4). People with disability living in urban slums also scored lower in the psychological and environmental domains than did people without. Comparisons within the group of people with disability (Table 5) identified differences in the environmental domain, with those living in non-slum urban areas scoring higher than those living in urban slum and rural areas. Comparisons between educational levels within the group of people with disability (Table 4) identified differences in all four domains; those who had not attended school had the lowest scores and those who had attended school for more than 10 years had the highest scores in all four domains.

**Table 5:** Comparisons of quality of life in the group with disability by gender, income, area of residence, and education

	Physical health				Psychological				Social relationships				Environmental			
	mean (95% CI)	mean (95%CI)	mean (95%CI)	P value	mean (95% CI)	mean (95% CI)	mean (95% CI)	P value	mean (95% CI)	mean (95% CI)	mean (95% CI)	P value	mean (95% CI)	mean (95% CI)	mean (95% CI)	P value
Gender																
Men with disability, *n* = 79 women with disability, *n* = 76	14.57 (13.96 to 15.18)	14.08 (13.41 to 14.77)		.292	14.57 (14.00 to 15.13)	14.15 (13.58 to 14.86)		.548	14.72 (13.88 to 15.56)	14.52 (13.58 to 15.45)		.946	13.18 (12.63 to 13.74)	12.45 (11.87 to 13.15)		.162
Income levels																
Group with disability with regular income, *n* = 63 without no or irregular income, *n* = 92	**15.17 (14.56 to 15.79)**	**13.68 (13.14 to 14.37)**		.003*	**15.25 (14.68 to 15.82)**	**13.75 (13.24 to 14.38)**		.002*	**15.82 (15.02 to 16.61)**	**13.78 (12.92 to 14.65)**		.002*	**13.82 (13.14 to 14.39)**	**12.14 (11.63 to 12.75)**		<.001*
Area of residence																
Group with disability non-slum urban, *n* = 38 urban slum, *n* = 63 rural, *n* = 54	15.01 (14.06 to 15.95)	13.67 (13.01 to 14.55)	14.50 (13.81 to 15.18)	.057	15.04 (14.31 to 15.76)	13.95 (13.33 to 14.73)	14.37 (13.62 to 15.13)	.063	15.46 (14.37 to 16.53)	14.39 (13.25 to 15.54)	14.28 (13.32 to 15.25)	.348	**13.58 (12.74 to 14.42)**	**12.17 (11.47 to 12.98)**	**13.06 (12.46 to 13.65)**	.026*
Education																
Group with disability no school, *n* = 22 school ≤10 years, *n* = 50 school > 10 years, *n* = 49	**13.81 (12.28 to 15.38)**	**14.49 (13.88 to 15.39)**	**15.78 (15.17 to 16.39)**	.024*	**12.90 (11.54 to 14.28)**	**14.36 (13.68 to 15.27)**	**15.76 (15.17 to 16.39)**	<.001*	**12.15 (10.50 to 13.81)**	**15.34 (14.36 to 16.33)**	**16.76 (16.06 to 17.46)**	<.001*	**12.02 (10.64 to 13.41)**	**12.97 (12.34 to 13.81)**	**13.99 (13.46 to 14.52)**	.010*

The lowest QOL scores (i.e., below 13) were found in the environmental domain for women with disability and no regular income who lived in urban slums and had not attended school and in the psychological and social relationship domains for people with disability who had not attended school (Table 5).

### Variables associated with low QOL

Table 6 presents results of logistic regression analysis, indicate prediction of lower QOL in three domains; Physical health, Psychological, and Environmental for persons with physical disability versus persons without disability. The association remain only in the Physical health when adjusting for gender, income, area of residence, and education. Results of univariate logistic regression analyses divided by subgroups and people with and without disability are presented in Table 6. The subgroups significantly (*p* < 0.05) associated with a higher risk of low physical health QOL were, in those with disability with no or irregular income, those living in urban slums and with no or < 10 years of schooling and, in the group without disability, women versus men. The subgroups significantly associated (*p* < 0.05) with a higher risk of low psychological QOL were, in those with disability and no or irregular income, those living in urban slums or rural areas and those with no or < 10 years of schooling. The subgroup significantly associated (*p* < 0.05) with a higher risk of low social relationship QOL were, in those with disability with no or irregular income and those with no or with < 10 years schooling. Finally, the subgroups significantly associated (*p* < 0.05) with a higher risk of low environmental QOL were, in the group with disability and no or irregular income, those living in urban slums and those with no or < 10 years of schooling (Table 7).

**Table 6:** Logistic regression indicating predicted lower quality of life (QOL) in the physical, psychological, and environmental domain in people with physical disability versus people without

	People with vs. without disability			
	Univariate		Adjusted**	
	OR	95% CI	OR	95% CI
Physical health QOL	3.54*	**3.54 to 5.90**	2.23*	**1.26 to 3.93**
Psychological QOL	1.80*	**1.80 to 2.95**	1.21	0.69 to 2.11
Social relationship QOL	1.37	1.37 to 2.22	0.80	0.46 to 1.39
Environmental QOL	1.91*	**1.91 to 3.08**	1.30	0.77 to 2.22

*Significant (*p* < 0.05)

**Adjusted for gender, income, area of residence, and education

**Table 7:** Logistic regression indicating which subgroups of people with disability are associated with lower quality of life (QOL) in the four domains included in the WHOQOL

		Physical health QOL		Psychological QOL		Social relationship QOL		Environmental QOL	
		OR	95% CI	OR	95% CI	OR	95% CI	OR	95% CI
Without disability	Female vs. male	2.68*	**1.16 to 6.20**	1.53	0.71 to 3.30	1.39	0.67 to 2.88	1.13	0.55 to 2.33
With physical disability	Female vs. male	1.50	0.79 to 2.85	1.26	0.67 to 2.36	0.75	0.40 to 1.41	1.58	0.83 to 3.00
Without disability	No or irregular income vs. regular income	0.94	0.42 to 2.12	1.37	0.64 to 2.94	0.77	0.37 to 1,61	1.10	0.53 to 2.29
With physical disability	No or irregular income vs. regular income	2.10*	**1.09 to 4.03**	2.67*	**1.37 to 5.23**	2.32*	**1.20 to 4.49**	2.80*	**1.44 to 5.43**
Without disability	Rural vs. non-slum urban	0.68	0.26 to 1.82	0.62	0.24 to 1.57	2.36	0.95 to 5,83	1.53	0.63 to 3.68
	Urban slum vs. non-slum urban	0.85	0.32 to 2.26	0.69	0.27 to 1.75	1.21	0.47 to 3,10	0.77	0.31 to 1.94
With physical disability	Rural vs. non-slum urban	1.92	0.82 to 4.45	4.04*	**1.57 to 10.39**	1.85	0.79 to 4,31	1.78	0.77 to 4.13
	Urban slum vs. non-slum urban	3.30*	**1.42 to 7.63**	4.69*	**1.86 to 11.82**	1.66	0.73 to 3,78	3.30*	**1.42 to 7.63**
Without disability	Attended ≤10 years vs. attended > 10 years	1.38	0.60 to 3.16	0.88	0.40 to 1.95	1.13	0.53 to 2.41	1.23	0.58 to 2.61
	Not attended vs. attended > 10 years	1.25	0.22 to 7.08	0.80	0.14 to 4.46	1.23	0.25 to 5.96	1.23	0.25 to 5.96
With physical disability	Attended ≤10 years vs. attended > 10 years	4.08*	**1.76 to 9.47**	4.81*	**1.94 to 11.98**	3.79*	**1.52 to 9.43**	3.09*	**1.36 to 7.04**
	Not attended vs. attended > 10 years	3.00*	**1.06 to 8.52**	9.52*	**3.01 to 30.15**	11.85*	**3.63 to 38.75**	4.42*	**1.50 to 12.98**

*Significant (*p* < 0.05)

## Discussion

This study shows that prosthetic and orthotic user had lower QOL scores in the physical health, psychological, and environmental domains than did those without disability. There were no significant differences in general QOL or general health between the groups with and without disability. The results also identified the subgroups of persons with disability with the lowest QOL scores that need to be prioritized in health, rehabilitation, and development programmes to achieve equity. These results correspond to the results of a systematic literature review [5] and to a case-control study of leprosy patients in India using WHOQOL-Bref [2], which both found lower QOL scores in people with disability in the physical health and psychological domains than in the control group. The most common cause of disability among the participants was poliomyelitis, which leaves survivors with reduced muscle strength and varying degrees of paralysis requiring knee-ankle-foot orthosis. In 2014, India was certified polio-free, yet many people continue to live with disabilities caused by polio [23]. To the best of our knowledge, only one other study has been published comparing QOL in a group of lower-limb amputees with that in the general population of India [6]. Therefore, the discussion that follows about concerns previous research conducted in other relevant countries.

A study of QOL using WHOQOL-Bref among Nigerians with unilateral lower-limb amputations [24] found a slightly higher overall QOL (i.e., 15.5) than did the present study (14.4). The group wearing prostheses in Nigeria [24] scored higher in the physical health, psychological, and environmental domains than did those with a unilateral lower-limb amputation not wearing prostheses. Women and those who had not yet received assistive devices needed attention, as their scores indicated lower QOL [24]. In a QOL study of lower-limb amputees conducted in Mumbai, India, using the 36-Item Short Form Health Survey (SF-36) [6], the physical and mental scores were significantly lower among amputees than among the general population. One Turkish study [25] comparing patients with post-polio syndrome and a control group without post-polio syndrome reported lower QOL in physical mobility, pain, and energy for post-polio patients [25].

### QOL in prosthetic and orthotic users in relation to gender

Our study found no significant differences between women and men with disability. This is in line with a systematic literature review [5], which found that only 3 of the 26 included studies in people with amputations reported significantly lower QOL among women than men.

However, men with disability had significantly lower QOL than men without in the physical health and environmental domains. This corresponds well with a finding regarding male leprosy patients in India [2]. In our study, women with disability had lower QOL than women without in the physical health domain, in contrast to the results of another Indian study [2], in which female leprosy patients’ scores indicated lower QOL than the female reference group’s in the psychological domain. One explanation for this difference could be variations in samples or types of disability. Nigerian women with lower-limb amputations also had lower QOL scores than men with lower-limb amputations in the physical health and social relationship domains [24].

### QOL for prosthetic and orthotic users in relation to income, education, and area of residence

Our results indicated that the subgroups that had no or irregular income and had not attended school were associated with a higher risk of lower QOL in all four domains: physical health, psychological, social relationships, and environment. Similar to this result, a Cambodian study [14] of people with disability reported that higher levels of education and higher contributions to household income were linked to higher QOL. Cambodian respondents who never attended school reported lower QOL than did respondents who had attended high school. Respondents in that study who did not contributing to the household income reported lower QOL in most domains [14]. A Thai study [3] obtained similar results when examining the health-related QOL (HRQOL) of people with a unilateral lower-limb amputation. Their results indicated that higher education and employment after an amputation were factors related to good HRQOL. A Bangladesh study investigating participants with ambulatory impairments found that use of assistive technology, such as wheelchairs, was positively associated with reduced poverty [26]. Our results also indicate that the subgroup living in slums had a higher risk of lower QOL in the physical health, psychological, and environment domains.

The Indian study [6] using SF 36 identified factors that affect QOL in lower-limb amputees reported that employment status, use of an assistive device, use of a prosthesis, co-morbidities, phantom limb pain, and residual stump pain significantly predicted both Physical and Mental HRQOL. The systematic review [5] of QOL studies of amputees found that most studies lacked information about background variables, such as education, employment, economic, and marital status. However, an another systematic review [27] found that higher physical activity, years of education, higher phantom pain severity, duration of phantom pain, level of amputation, and back pain were factors influencing HRQOL in American amputees caused in Vietnam and Iraq wars.

#### Groups vulnerable to low QOL

Our results indicate lower physical health QOL in prosthetic and orthotic users living in urban slums and rural areas than in those without disability living in these areas. This could be because daily life requires more physical activity in the rural areas and urban slums of India than in the non-slum urban areas. However, in general, limitations in physical functioning negatively affect amputees’ QOL [5]. People with disability without education or regular income are a vulnerable group that needs to be considered in CBR programmes [11, 12] and in planning rehabilitation services. The present study also found that people in urban slums with disability, no or irregular income, and no schooling had the lowest scores in all four QOL domains; to achieve equity, these groups also need to be included and prioritized in CBR activities and other development programmes, as well as in health and rehabilitation services.

### Limitations and methodological considerations

The present results are based on participants recruited from locations where Mobility India had established CBR programmes in slums and rural areas, which needs to be considered when interpreting the results. We suggest that those with physical disability who are not covered by CBR programmes and have not received services at all might experience an even greater negative QOL. The data were collected in different settings (at the rehabilitation centre, the participants’ homes, or in public) and these different environments may have affected the participants’ responses. However, all participants had similar access to CBR and centralized rehabilitation services at Mobility India; therefore, we think the different settings did not influence the results. Both the English and the Kannada versions of the WHOQOL-Bref were used. Researchers with experience in previous studies did the Kannada language translation [20, 21] in collaboration with the WHOQOL group. There may have been some differences in the way the messages were conveyed, but authors did not identify any problems with the translation during either data collection or data analysis.

The WHOQOL-Bref instrument used here involves the operationalization of the WHO theoretical construct of QOL in defined variables and the application of a survey methodology to quantify these variables. Furthermore, the strength of this instrument is the comprehensive validation performed in many countries [18, 22]. However, in this study, 38% of the participants (105 of a total of 277) did not answer the question “How satisfied are you with your sex life?” and several participants felt uncomfortable answering this question, resulting in low internal consistency (Cronbach’s α = 0.57) in the social relationship domain. This is important to consider when comparing the results for this domain between different age groups, sexes, and cultural contexts. In India, sex education is uncommon; people with disability are often perceived to lack the same desire as others, and they are aware of their inequality [28].

### Implications of QOL in relation to CBR programmes

Based on our results, access to education and income need to be prioritized to improve QOL in India for people with and without disability. We recommended that CBR and other development programmes for people with disability prioritize those who have no or irregular income, live in urban slums, and have not attended school. People with disability who have received rehabilitation services still need increased access to education, enough money to meet their basic needs, and information for everyday living to further improve their QOL. Opportunities for leisure activities have the potential to improve QOL in those with and without disability, especially those living in urban slums. In addition, a case study from India [29], a study from Iran [30], and a systematic review [31] all show that CBR interventions are important for vulnerable people with disability.

## Conclusion

People with physical disability have lower QOL in the physical health, psychological, and environmental domains than those without disability. Duration of education was positively associated with physical health, psychological, social relationship, and environmental QOL. People with disability who had not attended school had the lowest QOL score in the psychological domain. Income was associated with psychological and environmental QOL, while physical disability was associated only with physical health QOL. People with disability who have no or irregular income, live in urban slums, and have not attended school need to be prioritized in health, rehabilitation, and development programmes to achieve equity, as these groups reported the lowest QOL.

## Abbreviations

CBR: Community-based rehabilitation
HRQOL: Health-related quality of life
QOL: Quality of life
SF-36: 36-Item Short Form Health Survey
WHO: World Health Organization
WHOQOL-Bref: World Health Organization Quality of Life-BREF
